# Comprehensive investigation of a novel and safe lytic phage vB_AroA_KFSA9 as a commercial candidate for biocontrol of *Aeromonas hydrophila* on fresh produce

**DOI:** 10.1016/j.crfs.2025.101282

**Published:** 2025-12-18

**Authors:** In Young Choi, Su-Hyeon Kim, Mi-Kyung Park

**Affiliations:** a School of Food Science and Biotechnology, and Food and Bio-industry Research Institute, Kyungpook National University, Daegu, 41566, Republic of Korea; b Department of Infectious Disease Healthcare, Kyungpook National University, Daegu, 41566, Republic of Korea

**Keywords:** *Aeromonas hydrophila*, Lytic phage, Efficient biocontrol agent, Regulatory standards, Bactericidal effect, Fresh produce, Commercial candidate

## Abstract

*Aeromonas hydrophila* is an emerging foodborne pathogen, particularly prevalent in fresh produce, posing a risk to public health. Despite its significance, no commercial phage products have yet been developed to target *Aeromonas* spp. for food applications. This study characterized and evaluated a novel, safe *A. hydrophila*-specific phage, vB_AroA_KFSA9 as a commercial biocontrol candidate for application on fresh produce. vB_AroA_KFSA9, isolated from a poultry processing plant, exhibited high specificity only against nine *A. hydrophila* strains, showing no lytic activity against 4 *Aeromonas* species, 3 probiotic species, and 19 species of foodborne pathogen. Comprehensive genomic analysis confirmed the safety of the phage by revealing no undesirable genes associated with antibiotic resistance, bacterial virulence, or lysogeny. Furthermore, it identified vB_AroA_KFSA9 as a novel member of the genus *Teseptimavirus* within the family *Autotranscriptaviridae*, distinguished from related phages by a divergent tail fiber and unique endonuclease-associated genes. The phage remained stable even under food industry-relevant stress conditions, including pHs 4–10, 4–40 °C, and common sanitizers (acetic acid, citric acid, and hydrogen peroxide). The phage exhibited excellent bactericidal effects, achieving complete cell lysis within 3 h, even at a low multiplicity of infection (MOI) of 0.01. Applying vB_AroA_KFSA9 on *A. hydrophila*-contaminated cherry tomatoes and lettuce significantly reduced the number of *A. hydrophila* by ∼2.79 log CFU/cherry tomato and ∼2.45 log CFU/4 cm^2^ lettuce in 60 min, using an MOI of 1. These findings demonstrate vB_AroA_KFSA9 as a promising biocontrol candidate for future commercial application in the fresh produce chain.

## Highlights

• A novel, lytic vB_AroA_KFSA9 was stable under conditions relevant to fresh produce.
• Genomic analysis confirmed the novelty and safety of vB_AroA_KFSA9.
• vB_AroA_KFSA9 reduced *A. hydrophila* significantly on fresh produce.
• Bacterial regrowth suggests phage cocktails or hurdle strategies are needed.
• vB_AroA_KFSA9 is a biocontrol candidate against *A. hydrophila* on fresh produce.

## Graphical abstract

**Figure:**
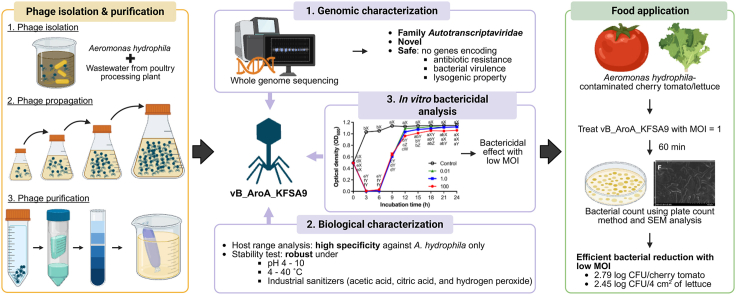

## 1 Introduction

*Aeromonas* spp. are rod-shaped, gram-negative, motile, non-spore-forming, and facultative anaerobic bacteria (Daskalov, 2006; Janda and Abbott, 2010). They are widely recognized as waterborne pathogens (Jun et al., 2013; Stratev and Odeyemi, 2016). Among them, *A. hydrophila* is a key zoonotic pathogen, responsible for most (>85 %) human infections, owing to its ability to produce enterotoxins and cytotoxins (Stratev and Odeyemi, 2016). In recent decades, *A. hydrophila* has emerged as one of the most important foodborne pathogens threatening public health (Abo-Shama et al., 2025). The remarkable tolerance of *A. hydrophila* to harsh environmental stresses, such as starvation and osmotic conditions, enables its survival and persistence in diverse foods, including meat, poultry, fresh produce, and fish (Awan et al., 2018; Pianetti et al., 2008; Radu et al., 2003). With rising demand for fresh fruits and vegetables driven by health-conscious diets (Park et al., 2013), the prevalence of *A. hydrophila* in fresh produce are in the range of 26–41% (Stratev and Odeyemi, 2016). An *A. hydrophila* outbreak occurred in China in 2012 because of contaminated cucumber and wild heartleaf, and resulted in acute diarrhea in over 200 individuals (Zhang et al., 2012). Notably, *A. hydrophila* is a psychrophile that can grow at cold temperatures commonly used for storing fresh produce (Berrang et al., 1989). This ability greatly increases the risk of bacterial growth even under refrigeration conditions during distribution and storage, underscoring the need for effective control strategies. However, the inherent characteristics of fresh produce—minimal processing and reliance on low-temperature storage—limit the antimicrobial or sanitizing options for controlling contamination. Conventional chemical or physical control methods may cause adverse effects (Oh and Park, 2017). For instance, chemicals such as chlorine and hypochlorite may form potential carcinogens. Irradiation, the most common physical method, may cause color changes, tissue softening, or unpleasant odors (Kim et al., 2024; Sun et al., 2024).

To overcome these limitations, increased attention is given to natural antimicrobials, such as essential oils, bacteriocins, lysins, and bacteriophages (phages). Phages, natural predators of bacteria, exhibit several advantages as biocontrol agents, including excellent host specificity (i.e., lytic properties against the host without disrupting the beneficial microflora), natural abundance (10^31^ particles), and robustness under harsh environmental conditions (Kim et al., 2023; Lee et al., 2020). Owing to these advantages, phage products, such as ListShield™, EcoShield™, SalmoFresh™, CampyShield™, and ShigaShield™, have been approved by the United States Food and Drug Administration (FDA) for food applications, targeting foodborne pathogens including *Listeria monocytogenes*, *Escherichia coli* O157:H7, *Salmonella* spp., *Campylobacter jejuni*, and *Shigella sonnei* (Jagannathan et al., 2022). To date, there are no commercial phage products targeting *Aeromonas* spp. for food applications. BAFADOR® is the only product available, limited to use in animal feed. The development of phage products for food applications requires regulatory approval, such as Generally Recognized As Safe (GRAS) status, to ensure both safety and efficacy. To meet these standards, the phage must comply with safety criteria, including inability to transfer genetic material and absence of genes encoding virulence factors, pathogenicity factors, or antibiotic resistance (Cui et al., 2024). Furthermore, the application feasibility depends on the phage concentration required for effective control (Lewis and Hill, 2020). For example, commercial phage products, such as SalmoFresh™, Listex™ P100, ShigaShield™, and ListShield™, typically require multiplicity of infection (MOI) values of 10^2^–10^3^ to achieve substantial bacterial reductions (Lewis and Hill, 2020; Soffer et al., 2017; Zhang et al., 2019). These considerations highlight that future *A. hydrophila*-specific phage products should meet GRAS requirements encompassing both safety and efficacy, while exhibiting high performance to ensure practical and cost-effective application.

Therefore, this study focused on a comprehensive investigation of *A. hydrophila*-specific phages, aiming to develop safe and effective candidates for commercial application. In line with the requirements for regulatory approval, the isolated phage was evaluated in terms of its host range, stability under different conditions, *in vitro* bactericidal effect, and genetic safety. Moreover, this study demonstrated the practical applicability of the phage by validating its efficacy at low MOIs on *A. hydrophila*-contaminated cherry tomatoes and lettuce, thereby addressing a critical gap in previous phage biocontrol studies.

## 2 Materials and methods

### 2.1 Bacterial strains and growth conditions

Table 1 lists the bacterial strains (*n* = 43) used in this study. Bacterial strains except probiotic strains were grown aerobically in 25 mL of tryptic soy broth (TSB; Becton, Dickinson and Company (BD), Franklin Lakes, NJ, USA) with constant agitation at 37 °C for 16 h. Probiotics including *Bifidobacterium breve*, *Lactiplantibacillus plantarum*, and *Limosilactobacillus reuteri* were cultured at 37 °C in de Man, Rogosa, and Sharpe medium (BD) under hypoxic conditions (5 % CO_2_ and 2 % O_2_). *A. hydrophila* ATCC 7966 was used as an indicator strain for phage isolation. All other strains were used for the host range study. Each overnight culture was centrifuged at 2400×*g* for 4 min and washed with sterilized phosphate-buffered saline (PBS; pH 7.4, Life Technologies Co., Carlsbad, CA, USA). This step was repeated three times. The final concentration of each bacterial suspension was adjusted to 8 log colony-forming units (CFU)/mL, using a pre-constructed standard curve based on optical density (OD) measured at 600 nm.

**Table 1:** Host range and efficiency of plating of vB_AroA_KFSA9.

Bacterial strains^a^	EOP^b^
*Aeromonas hydrophila* ATCC 7966	1.00 ± 0.00
*A. hydrophila* SNUFPC A3	0.98 ± 0.02
*A. hydrophila* SNUFPC A5	0.92 ± 0.10
*A. hydrophila* SNUFPC A6	1.00 ± 0.08
*A. hydrophila* SNUFPC A7	0.87 ± 0.05
*A. hydrophila* SNUFPC A8	0.90 ± 0.07
*A. hydrophila* SNUFPC A9	1.00 ± 0.00
*A. hydrophila* SNUFPC A10	0.95 ± 0.11
*A. hydrophila* SNUFPC A11	1.00 ± 0.00
*A. salmonicida* ATCC 33658	-c
*A*. *media* ATCC 33907	–
*A. sobria* ATCC 43979	–
*A. veronii* ATCC 9071	–
*Bacillus cereus* ATCC 13061	–
*B. subtilis* ATCC 6633	–
*B. subtilis* IDCC 1101	–
*Bifidobacterium breve* IDCC 4401	–
*Escherichia coli* O157:H7 ATCC 43895	–
*E. coli* ATCC BAA-2196	–
*E. coli* K12 VSM 1692	–
*Klebsiella pneumoniae* ATCC 13883	–
*Lactiplantibacillus plantarum* IDCC 3501	–
*Limosilactobacillus reuteri* IDCC 3701	–
*Listeria monocytogenes* ATCC 1911	–
*L. monocytogenes* ATCC 7644	–
*L. innocua* ATCC 33090	–
*Pseudomonas aeruginosa* ATCC 9027	–
*Salmonella* Enteritidis ATCC 13076	–
*S.* Dublin NCCP 13700	–
*S.* Heidelberg NCCP 13698	–
*S.* Montevideo NCCP 13704	–
*S.* Newport NCCP 13686	–
*S.* Panama NCCP 13694	–
*S.* Typhimurium ATCC 19586	–
*Shigella flexneri* 2457T	–
*S. sonnei* ATCC 9290	–
*Staphylococcus aureus* ATCC 25923	–
*Vibrio parahaemolyticus* ATCC 17802	–
*Yersinia enterocolitica* ATCC 23715	–

a ATCC, American Type Culture Collection; SNUFPC, natural isolates (Han et al., 2012) obtained from College of Veterinary Medicine and Research Institute for Veterinary Science, Seoul National University; IDCC, Strains obtained from Ildong Bioscience Co., Ltd. (Pyeongtaek, Republic of Korea); NCCP, National Culture Collection for Pathogens.

b EOP, efficiency of plating.

c –, no clear zone formation.

### *2.2* Isolation, propagation, and purification of an *A. hydrophila*-specific phage from a poultry processing plant

Sixteen wastewater samples were collected from slaughterhouses, poultry processing plants, and fish farms. The *A*. *hydrophila*-specific phage was isolated, propagated, and purified from the samples, following a previously described protocol (Choi et al., 2020). Briefly, 25 mL of each sample was incubated with 225 mL of TSB containing 1 mL of the indicator strain at 37 °C for 16 h. After centrifugation at 4000×*g* for 10 min, the supernatant was filtered using a 0.20 μm cellulose acetate filter (Toyo Roshi Kaisha Ltd., Tokyo, Japan). The filtrate was subjected to a plaque assay for single phage isolation. For this, 100 μL of the serially diluted filtrate and 200 μL of the indicator strain were added to 4 mL of TA soft agar (4 g of agar, 8 g of nutrient broth, 5 g of NaCl, 0.2 g of MgSO_4_, 0.05 g of MnSO_4_, and 0.15 g of CaCl_2_ per liter) before pouring it onto a tryptic soy agar (TSA; BD, Franklin Lakes, NJ, USA) plate and incubating at 37 °C for 16 h. High-titer propagation of the single phage (referred to as vB_AroA_KFSA9 hereafter) was conducted by incubating 1 % (v/v) of the indicator strain (8 log CFU/mL) in TA broth at 37 °C for 2 h, with gentle agitation, followed by infection with vB_AroA_KFSA9. After further incubation at 37 °C for 2 h, the culture was centrifuged at 2400×*g* and 4 °C for 10 min and filtered. The propagation procedure was repeated by gradually increasing the culture volume (up to 3 L) to assess batch-to-batch consistency in phage production, aiming for yields exceeding 10–11 log plaque-forming units (PFU)/mL. The final filtrate of the propagated phage was subjected to polyethylene glycol (PEG) precipitation, followed by ultrafiltration with an Amicon® ultra centrifugal filter (100 kDa, Millipore Sigma Co., Burlington, MA, USA), CsCl density-gradient ultracentrifugation, and dialysis in SM buffer (Kim et al., 2023) to obtain a highly purified phage and reduce the endotoxin concentration in the phage suspension. The concentration of vB_AroA_KFSA9 was measured at every step, using the plaque assay.

### 2.3 Host range and efficiency of plating of vB_AroA_KFSA9

The host range of vB_AroA_KFSA9 was assessed using a dot assay. Briefly, 10 μL of vB_AroA_KFSA9 (8 log PFU/mL) was dotted on the surface of pre-solidified TA soft agar containing 200 μL of each bacterial strain (8 log CFU/mL) listed in Table 1, and incubated at 37 °C for 16 h. After identifying vB_AroA_KFSA9-susceptible bacterial strains by the formation of a clear zone, the efficiency of plating (EOP) was determined via plaque assay. The EOP value was calculated by dividing the number of plaques on each tested strain by the number of plaques on the indicator strain (Choi et al., 2020).

### 2.4 Genome extraction, sequencing, and bioinformatics analyses of vB_AroA_KFSA9

The genomic DNA of vB_AroA_KFSA9 was extracted and purified using a phage DNA isolation kit (Norgen Biotek Corp., Thorold, ON, Canada). Whole-genome sequencing of the purified DNA was performed (LabGenomics Co., Seongnam, Republic of Korea) using the Illumina platform (Illumina Inc., San Diego, CA, USA). The raw reads were trimmed using Trimmomatic (Illumina) to eliminate low-quality reads and adapter sequences. The *de novo* assembly of sequences was performed using various k-mers with the SPAdes genome assembler (Illumina). The open reading frames (ORFs) of the assembled sequence were predicted and annotated using the Rapid Annotations using Subsystems Technology (RAST) server (Brettin et al., 2015) and Pharokka pipeline (v1.7.9) (Bouras et al., 2023). The vB_AroA_KFSA9 genome was screened using the VirulenceFinder pipeline (v3.2.0) and the allergen database (http://www.allergenonline.com) from Food Allergy Research & Education (FARE) to confirm the genes associated with virulence and allergenic factors, respectively. Furthermore, the phage genome was checked against the Comprehensive Antibiotic Resistance Database (CARD) (Alcock et al., 2023) and ResFinder 4.1 for antimicrobial resistance genes. Finally, a genome map was generated using SnapGene (GSL Biotech LLC Co., San Diego, CA, USA). The phage lifestyle was classified using the PhageAI platform (https://phage.ai/), and its phylogenetic tree was constructed using the Virus Classification and Tree Building Online Resource (VICTOR) with the d0 formula (Meier-Kolthoff and Göker, 2017) and iTOL (https://itol.embl.de). The average nucleotide identity (ANI) between vB_AroA_KFSA9 and its close relatives was calculated using the FastANI pipeline (v1.33) (Jain et al., 2018), with default parameters. Finally, the genome was compared with those of the closest phages via Clinker (v0.0.3) with a 0.7 identity parameter (Gilchrist and Chooi, 2021). The complete genome sequence of the phage was deposited in the GenBank database under nucleotide sequence accession number PQ824399.

### 2.5 Stability analysis of vB_AroA_KFSA9

The stability of vB_AroA_KFSA9 was investigated by exposing it to various pHs, temperatures, and sanitizers. The effect of pH on the phage was assessed by incubating a mixture of 100 μL of vB_AroA_KFSA9 (8 log PFU/mL) and 900 μL of SM buffer, previously adjusted to a pH range of 3–11 at 37 °C for 1 h. The effect of temperature was evaluated by incubating the same amount of phage with SM buffer (pH 7.6) at various temperatures (4, 10, 20, 30, 40, 50, 60, and 70 °C) for 1 h. To investigate the stability of vB_AroA_KFSA9 when exposed to sanitizers, the same amount of phage was mixed with 900 μL of each of the following sanitizer: 35 % hydrogen peroxide (food grade, Hansol Chemical Co., Seoul, Republic of Korea), 10 % acetic acid (Sigma-Aldrich Co., St. Louis, MO, USA), and 10 % citric acid (Sigma-Aldrich Co.). Following 1 h of exposure to each condition, the stability of vB_AroA_KFSA9 was determined by measuring its titer using the plaque assay.

### *2.6* *In vitro* bactericidal analysis of vB_AroA_KFSA9

To investigate the bactericidal activity of vB_AroA_KFSA9 *in vitro*, 1 mL of an *A. hydrophila* ATCC 7966 suspension (8 log CFU/mL) was inoculated into 20 mL of TSB. Various concentrations of phage suspension (100 μL) were added to each tube containing bacterial suspension to obtain MOIs of 0.01, 1, and 100. Samples were collected at 3 h intervals during incubation at 37 °C, and their OD were measured at 600 nm over 24 h (Haq et al., 2012).

### 2.7 Application of vB_AroA_KFSA9 to cherry tomatoes and lettuce

Cherry tomatoes and lettuce were purchased from a local grocery store, and the lettuce was cut into 2 × 2 cm^2^ pieces. The samples were soaked in a chlorine solution (200 ppm) for 10 min, rinsed three times with sterilized distilled water, and exposed to ultraviolet (UV) light for 30 min in a safety cabinet to eliminate background microorganisms (Snyder et al., 2016). To evaluate the bactericidal effect of vB_AroA_KFSA9, the fresh produce samples were dipped in 200 mL of an *A. hydrophila* ATCC 7966 suspension (8 log CFU/mL). Based on the optimization of bacterial attachment time (Fig. S2), the samples were incubated for 30 min before phage treatment. Each sample contaminated with *A*. *hydrophila* was dipped in 200 mL of vB_AroA_KFSA9 at an MOI of 1 or PBS, and incubated at 22 °C for 30, 60, 90, and 120 min. Each sample was transferred to a stomacher bag containing 180 mL of PBS and homogenized at 120 rpm for 2 min using stomacher (Hansol Tech, Co., Seoul, Republic of Korea). The pH of each homogenate was adjusted to 7.0 before plating to eliminate any pH-related effect on bacterial growth. Surviving *A. hydrophila* were enumerated by plating on *Aeromonas*-selective agar (HiMedia Laboratories Pvt Ltd., India). Bacterial attachment on the cherry tomato surface was visualized using a scanning electron microscope (FE-SEM Hitachi SU-8220, Hitachi Co., Japan) at 5 kV after treatment with osmium tetroxide (Electron Microscopy Sciences, Hatfield, PA, USA). Attached cells were counted using the ImageJ software (National Institutes of Health, USA) at a magnification of 5000×*g*.

### 2.8 Statistical analysis

All experiments were performed at least triplicate, and the data are expressed as the mean ± standard deviation. GraphPad Prism v.10 (GraphPad, San Diego, CA, USA) was used for statistical analyses. Students’ unpaired *t*-test and one-way analysis of variance (ANOVA) were used for comparisons between two groups and among more than two groups, respectively.

## 3 Results

### 3.1 Isolation and purification of *A. hydrophila*-specific phages

A phage infecting *A. hydrophila* was isolated from wastewater from a poultry processing plant. This phage exhibited potent lytic activity against *A. hydrophila*, with a distinctive clear zone (1.60 ± 0.14 cm in diameter) (Fig. S1). Thus, the phage was purified, propagated at a final concentration of 9.4 log PFU/mL, and named vB_AroA_KFSA9.

### 3.2 Host range and efficiency of plating of vB_AroA_KFSA9

As a first step in evaluating the potential application of vB_AroA_KFSA9 as a biocontrol agent, its host range was investigated against 5 *Aeromonas species*, 3 probiotic species, and 19 major foodborne pathogens (Table 1). vB_AroA_KFSA9 showed lytic activity only against *A. hydrophila* strains (ATCC 7966, SNUFPC A3, SNUFPC A5, SNUFPC A6, SNUFPC A7, SNUFPC A8, SNUFPC A9, SNUFPC A10, and SNUFPC A11) with high plaque production (EOP value > 0.5) (Khan Mirzaei and Nilsson, 2015; Lee et al., 2020), while no lytic activity against 4 other *Aeromonas species*, 3 probiotic species, or 19 species of foodborne pathogens. This result demonstrated that vB_AroA_KFSA9 has a narrow host range against *A. hydrophila* only. Such narrow host range suggests its suitability as a targeted biocontrol agent against *A. hydrophila*.

### 3.3 Genomic characteristics of vB_AroA_KFSA9

**Fig. 1:**
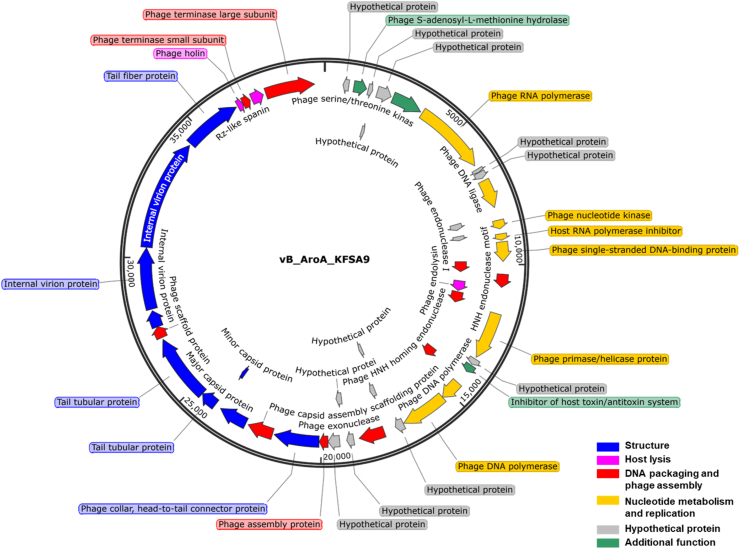
Circularized genome map of vB_AroA_KFSA9. The arrows represent the open reading frames (ORFs), and their colors indicate their functions: blue, phage structure; pink, host lysis; red, DNA packaging and phage assembly; grey, hypothetical protein; green, additional functions; yellow, nucleotide metabolism and replication.

The genome of vB_AroA_KFSA9 consisted of 39,785 bp double-stranded (ds) DNA with a GC content of 53.1 % (Fig. 1). The complete genome encoded 48 predicted ORFs (33 functional and 15 hypothetical ORFs). The functional ORFs were classified into five categories: phage structure (nine ORFs, blue arrows), host lysis (three ORFs, pink arrows), DNA packaging and phage assembly (ten ORFs, red arrows), nucleotide metabolism and replication (eight ORFs, yellow arrows), and additional functions (three ORFs, grey arrows). The host lysis genes (indicated by pink arrows) included endolysin (*orf*18), holin (*orf*45), and spanin (*orf*47). PhageAI analysis further classified vB_AroA_KFSA9 as a lytic phage with 99.77 % probability. More detailed function and categorization of each annotated ORF are listed in Table S1. Notably, no genes related to antibiotic resistance, bacterial virulence, or lysogeny were detected based on genome annotation and database-based screening.

### 3.4 Phylogenetic and comparative analyses of vB_AroA_KFSA9

**Fig. 2:**
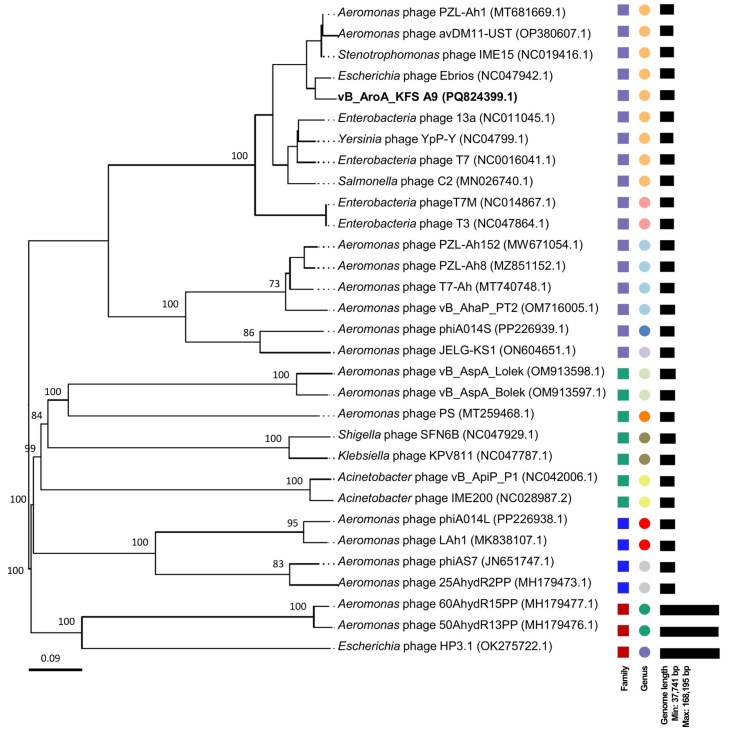
Phylogenetic analysis of vB_AroA_KFSA9 based on the complete genome. Members of the families *Autotranscriptaviridae*, *Autoscriptoviridae*, *Autonotataviridae*, and *Straboviridae* are indicated by the purple, green, blue, and red boxes, respectively. Circles with different colors represent different genera.

**Fig. 3:**
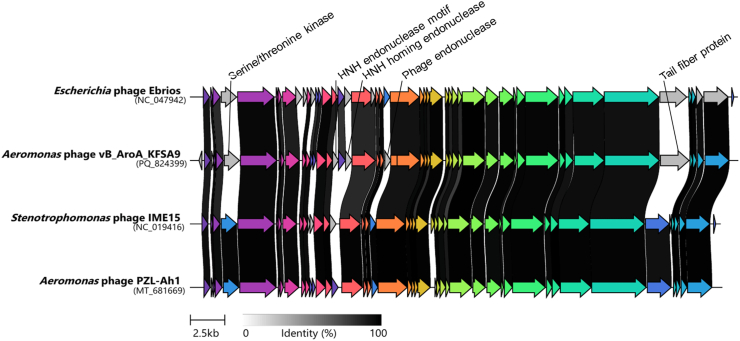
Comparative genomic analysis of vB_AroA_KFSA9 with closely related phages, including Ebrios, IME15, and PZL-Ah1. Coding sequences are represented by arrows, with colors reflecting homologous gene groups. Grey bars between genomes indicate pairwise amino acid identity, with darker shading representing higher similarity. Grey arrows represent coding sequences without detectable homologs among the compared genomes.

Phylogenetic analysis (Fig. 2) placed vB_AroA_KFSA9 within the family *Autotranscriptaviridae* (indicated by purple boxes), clustering in a single clade with phages belonging to the genus *Teseptimavirus* (indicated by orange circles). Within this genus, vB_AroA_KFSA9 exhibited the closest identity (>95 %) with four phages: IME15, PZL-Ah1, Ebrios, and avDM11-UST (Table S2). ANI values for IME15, PZL-Ah1, and Ebrios (Table S3) exceeded 95 %, which is commonly used as a species-level boundary in phage taxonomy (Turner et al., 2021). Given these close relationships, comparative genomic analysis was conducted with the most similar references, including IME15, PZL-Ah1, and Ebrios. As shown in Fig. 3, vB_AroA_KFSA9 shared the conserved genome organization of *Teseptimavirus* members, while displaying differences in the tail fiber (*orf*44) and multiple endonuclease-associated genes (*orf*17, *orf*19, and *orf*23).

### 3.5 Stability of vB_AroA_KFSA9

**Fig. 4:**
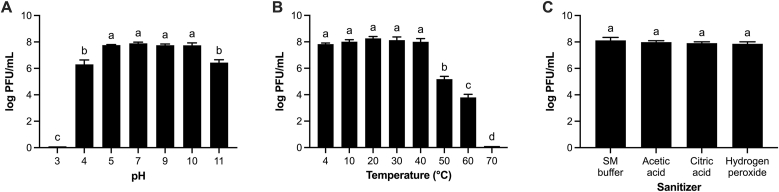
Stability of vB_AroA_KFSA9 exposed to various (A) pHs, (B) temperatures, and (C) sanitizers. The letters (a–d) on the bars indicate significant differences at *P <* 0.05 (*n* = 3, one-way ANOVA).

The stability of vB_AroA_KFSA9 was evaluated under various pH, temperature, and sanitizer conditions to assess its potential for practical use (Fig. 4). The phage remained stable at a wide pH range (4.0–10.0) but was completely inactivated at pH 3.0, with concentration decreasing to 0.10 ± 0.00 log PFU/mL (Fig. 4A) (*P* < 0.05). Furthermore, the phage concentration remained around 8 log PFU/mL at 4, 10, 20, 30, and 40 °C (Fig. 4B). However, exposure to 50 °C and 60 °C dropped the phage concentration to 5.18 ± 0.20 log PFU/mL and 3.79 ± 0.24 log PFU/mL, respectively (*P* < 0.05), and almost no phages remained at 70 °C (0.12 ± 0.00 log PFU/mL). Moreover, vB_AroA_KFSA9 exhibited robust stability against commonly used sanitizers such as acetic acid, citric acid, and hydrogen peroxide (Fig. 4C). These findings indicate that vB_AroA_KFSA9 remains stable under a range of conditions relevant to fresh produce processing, supporting its applicability as a biocontrol agent.

### *3.6* *In vitro* bactericidal effect of vB_AroA_KFSA9

**Fig. 5:**
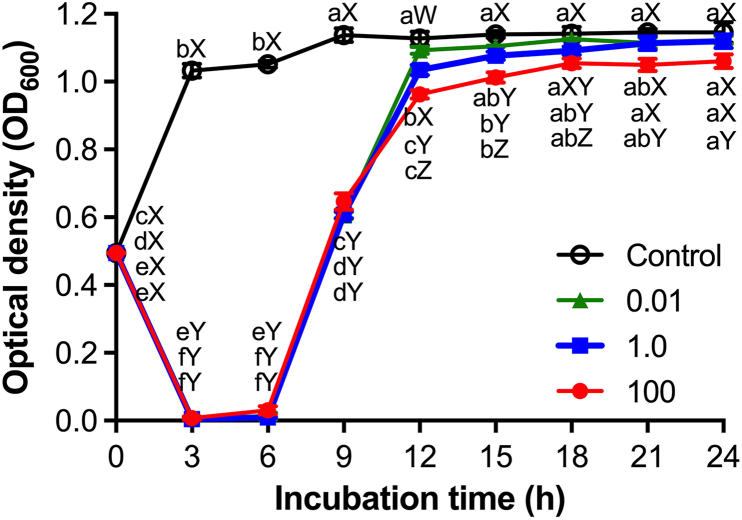
Bactericidal effect of vB_AroA_KFSA9 against *Aeromonas hydrophila* at various multiplicity of infection (MOI) values. The letters (a–f and W–Z) on the lines indicate significant differences within the same treatment and incubation time, respectively, at *P <* 0.05 (*n* = 3, one-way ANOVA).

The *in vitro* bactericidal effect of vB_AroA_KFSA9 was evaluated by monitoring bacterial growth over 24 h (Fig. 5). In the control group, the OD increased rapidly within 3 h and reached 1.15 ± 0.03 at 24 h. By contrast, all phage-treated groups exhibited a sharp reduction within 3 h at all MOIs, with no significant differences among MOIs. This bactericidal effect was sustained until 6 h post-treatment. Although bacterial regrowth was observed thereafter, with OD increasing to 0.96–1.13 after 12 h incubation, cultures treated at an MOI of 100 still remained at significantly lower OD than the control throughout the incubation period (*P* < 0.05). These results demonstrate that vB_AroA_KFSA9 controlled *A. hydrophila in vitro* rapidly and in a short period.

### 3.7 Bactericidal effect of vB_AroA_KFSA9 on *A. hydrophila*-contaminated cherry tomato and lettuce

**Fig. 6:**
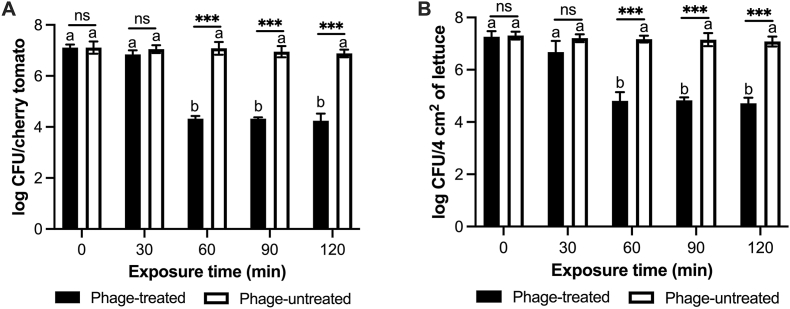
Bactericidal effect of vB_AroA_KFSA9 on (A) cherry tomatoes and (B) lettuce contaminated with *Aeromonas hydrophila*. The letters (a–b) indicate significant differences among incubation times at *P <* 0.05 (*n* = 3, one-way ANOVA); ns and ∗∗∗ indicate no significant differences (*P* > 0.05) and significant differences (*P* < 0.001), respectively, between the phage-treated and untreated groups (*n* = 3, Student's unpaired *t*-test).

**Fig. 7:**
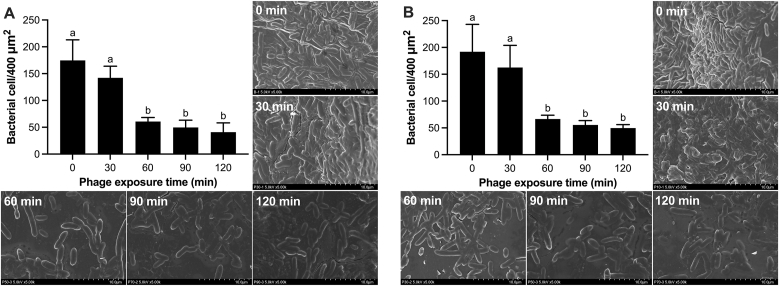
Density and SEM images of *Aeromonas hydrophila* on (A) cherry tomatoes and (B) lettuce, following treatment with vB_AroA_KFSA9. Representative SEM images are shown with incubation times. The letters (a–b) indicate significant differences among incubation times at *P <* 0.05 (*n* = 3, one-way ANOVA).

vB_AroA_KFSA9 was applied on the surface of cherry tomatoes and lettuce contaminated with *A. hydrophila* to evaluate the bactericidal effect of the phage (Fig. 6). Although no MOI-dependent differences were observed *in vitro*, an MOI of 1 was applied for food experiments to account for the expected reduction in phage efficacy on fresh produce surfaces. In the phage-untreated group, the bacterial concentration on both cherry tomatoes and lettuce did not change significantly during the incubation time (Fig. 6). By contrast, when vB_AroA_KFSA9 was applied on the surface of fresh produce, the bacterial concentration was sustained for the first 30 min and then significantly declined by ∼2.79 log CFU/cherry tomato (Fig. 6A) and by ∼2.45 log CFU/4 cm^2^ lettuce (Fig. 6B) at 60 min (*P* < 0.05). These reduced levels were maintained until 120 min, without regrowth. SEM analysis confirmed these reductions in the density of *A*. *hydrophila* on the surface of cherry tomatoes and lettuce (Fig. 7). On cherry tomatoes (Fig. 7A), bacterial density significantly decreased from 170 ± 38 cells/400 μm^2^ at 0 min to 60 ± 7 cells/400 μm^2^ after 60 min of phage exposure (*P* < 0.05). Similarly, lettuce samples showed a significant reduction from 192 ± 50 cells/400 μm^2^ to 66 ± 7 cells/400 μm^2^ after 60 min (Fig. 7B, *P* < 0.05). These results demonstrate that vB_AroA_KFSA9 reduces *A. hydrophila* contamination on cherry tomatoes and lettuce within 60 min with an MOI of 1.

## 4 Discussion

Despite growing concern for the presence of *A. hydrophila* in fresh produce, no phage products targeting *A. hydrophila* have yet been commercialized for food applications. To address this gap, vB_AroA_KFSA9 was isolated from a poultry processing plant and characterized according to the regulatory criteria for phage product approval for food applications. Host range analysis revealed that vB_AroA_KFSA9 exhibited a narrow host range, being effective only against nine *A. hydrophila* strains. The phage exhibited no lytic activity against four other *Aeromonas species*, three probiotic species, or 19 species of foodborne pathogens (Table 1). By contrast, the only available *Aeromonas*-phage product, BAFADOR®, was developed for feed additive rather than food and consists of three *A. hydrophila*-specific phages (25AhydR2PP, 50AhydR13PP, and 60AhydR15PP). Testing on *A. hydrophila*, *A. sobria*, *A. salmonicida*, and *Pseudomonas fluorescens* revealed that these phages displayed broad host ranges, with 25AhydR2PP infecting both *A. hydrophila* and *A. sobria*, and 50AhydR13PP and 60AhydR15PP infecting all three *Aeromonas species* (Kazimierczak et al., 2019; Schulz et al., 2019). Although a broad host range seems advantageous by covering multiple bacteria, it also carries the risk of reduced lysis efficacy (Molina et al., 2020). On the other hand, the narrow host range of vB_AroA_KFSA9 ensures highly selective activity against *A. hydrophila strains* and minimizes off-target effect on non-target bacteria, in line with requirements for demonstrating efficacy against the intended target.

From a genomic safety perspective, comprehensive annotation and screening revealed that the genome of vB_AroA_KFSA9 lacked any genes encoding antibiotic resistance, bacterial toxins, or lysogenic elements (Fig. 1 and Table S1). In addition, the presence of a canonical lytic module comprising a class II holin (*orf*45), spanin (*orf*47), and an endolysin (*orf*18) indicates that vB_AroA_KFSA9 possesses a strict lytic nature (Cahill and Young, 2019). These criteria are consistent with the requirements for the GRAS approval of commercial phage products, such as ListShield™ *(*Vikram et al., 2021*)*, SalmoFresh™ (Woolston et al., 2013), ShigaShield™ (Moye et al., 2018), and BAFADOR® (Kazimierczak et al., 2019). Therefore, the genomic safety of vB_AroA_KFSA9 was validated at a level comparable to that of existing commercial phage products, supporting its suitability for commercial development.

In addition to genomic safety, phylogenetic analysis (Fig. 2) placed vB_AroA_KFSA9 within the genus *Teseptimavirus* of the family *Autotranscriptaviridae*, a lineage with no previously reported *Aeromonas* phages according to the most recent ICTV classification (Turner et al., 2025). Moreover, comparative genomic analysis (Fig. 3) with related phages, including *Stenotrophomonas* phage IME15 (NC_019416.1), *Escherichia* phage Ebrios (NC_047942.1), and *Aeromonas* phage PZL-Ah1 (MT_681669.1), highlighted the unique features of vB_AroA_KFSA9 in the tail fiber (*orf*44) and multiple endonuclease-associated genes (*orf*17, *orf*19, and *orf*23). Given that tail fibers are key determinants of phage-host recognition and specificity (Latka et al., 2019), these differences in the tail fiber may explain its narrower host range, compared to the polyvalent phage PZL-Ah1 that infects both *A*. *hydrophila* and *A*. *veronii* (Yu et al., 2022a). The genomic safety and taxonomic novelty of vB_AroA_KFSA9 demonstrated its suitability as a new candidate for commercial phage products in food applications.

Since phages are exposed to diverse physicochemical conditions when applied to food, their stability under various conditions should be considered when evaluating their potential as biocontrol agents (Hou et al., 2023). vB_AroA_KFSA9 remained stable across pHs 4–10 (Fig. 4A) and 4–40 °C (Fig. 4B). This pH range overlapped with the growth range of *A. hydrophila* (pHs 4.5–9) (Igbinosa et al., 2012). Lytic activity was almost completely lost at pH 3, consistent with other *A. hydrophila*-specific phages in previous studies (Chandrarathna et al., 2020; Hou et al., 2023; Islam et al., 2021; Jun et al., 2013). The stable temperature range of vB_AroA_KFSA9 was consistent with those of other *A. hydrophila*-specific phages, including N21, W3, G65, Y71, and Y81 (Cao et al., 2020), VB_AhaP_PZL-Ah8 (Yu et al., 2022a), and AHPMCC7 (Ghosh et al., 2023). More importantly, the pH and temperature stabilities of the phage were comparable to those of GRAS-approved commercial phage products, such as Listex™ P100 (EFSA Panel on Biological Hazards, 2016) and ListShield™ (Henderson et al., 2019), which remained stable across pHs 4–10 and 2–42 °C. In addition, vB_AroA_KFSA9 was stable in the presence of 10 % solutions of acetic acid, citric acid, and hydrogen peroxide (Fig. 4C). Such concentrations greatly exceed those permitted for fresh produce washing (0.03–0.07 % acetic acid, 1.6–3.3 % citric acid, and 0.03–0.05 % hydrogen peroxide; 21 CFR 178.1010) and were intentionally used as worst-case challenge conditions. Thus, this observation suggests that vB_AroA_KFSA9 is likely to withstand the considerably milder conditions used in fresh produce sanitation, supporting its compatibility with existing washing protocols. Overall, the robustness of vB_AroA_KFSA9 under various environmental conditions underscores its feasibility as a commercial biocontrol candidate in the fresh produce chain.

*In vitro* bactericidal analysis of vB_AroA_KFSA9 (Fig. 5) showed clear lysis of *A*. *hydrophila* after exposure for 3 h, sustained for up to 6 h in an MOI-independent manner. Previous studies reported the bactericidal effects of *A*. *hydrophila*-specific phages. For instance, Yu et al. (2022b) reported that clear lysis of *A*. *hydrophila* was achieved only when a phage cocktail of vB_AhaP_PZL-Ah1 and vB_AhaP_PZL-Ah8 was applied at an MOI of 0.1, whereas individual phages showed limited lysis. By contrast, vB_AroA_KFSA9 alone achieved clear lysis of *A. hydrophila* even at a lower MOI of 0.01, underscoring its strong bactericidal potential without the need for a cocktail formulation. Compared with commercial phage products, such as ListShield™ and SalmoFresh™, which generally require high application doses (typically ≥ 10^8^ PFU/g or MOIs above 10^3^–10^4^), our phage exhibited high efficiency at a low MOI. Effects of vB_AroA_KFSA9 at low MOI may offer advantages for industrial-scale applications and cost-efficiency in future applications. However, bacterial regrowth was observed after 6 h of incubation following phage treatment. Similar bacterial regrowth has been reported in other phages and is linked to the emergence of phage-resistant mutants (Li et al., 2022). Bacteria have evolved multiple defense mechanisms to resist phage infection, including blocking phage DNA entry, degrading phage genomes using restriction-modification systems (Sneppen et al., 2015) and CRISPR-Cas systems (Al-Shayeb et al., 2020), preventing phage adsorption through receptor modification (Kintz et al., 2015), or abortive infection system limiting phage proliferation (Sekulovic et al., 2015). These mechanisms may have contributed to the short duration of bacterial control here, which may limit the application of vB_AroA_KFSA9. Recent studies have emphasized that integrating phages into hurdle technologies may help mitigate bacterial regrowth and resistance in practical applications (Liu et al., 2023). In this context, such a limitation could potentially be addressed by hurdle strategies, such as employing a phage cocktail (O'flynn et al., 2004; Tanji et al., 2004) or integrating with other methods, such as ultrasound (Yuan et al., 2025), chemical sanitizer application (Liu et al., 2023), irradiation (Myshkevych et al., 2025), and high-pressure processing (Jagannathan et al., 2022).

Cherry tomatoes and lettuce were selected for testing the phage on fresh produce because they are widely consumed in the United States, ranking among the top five most-consumed fresh produce, and have been frequently implicated in foodborne outbreaks (Kantor and Blazejczyk, 2020; Yang and Scharff, 2024). When applied to cherry tomato or lettuce contaminated with *A. hydrophila*, vB_AroA_KFSA9 significantly reduced bacterial numbers by ∼2.79 log CFU/cherry tomato (Fig. 6A) and ∼2.45 log CFU/4 cm^2^ lettuce (Fig. 6B) at 60 min post-treatment with an MOI of 1 (*P* < 0.05). Although comparable findings have not been reported for commercial *Aeromonas* phage products for food applications, Islam et al. (2021) found that ZPAH7 reduced 2.4 log CFU/cm^2^ and 2.6 log CFU/cm^2^ of *A. hydrophila* on lettuce, with MOIs of 100 and 1,000, respectively. Hou et al. (2023) used ZPAH34 and achieved 2.05 log CFU/cm^2^ and 1.46 log CFU/cm^2^ reductions on lettuce, with MOIs of 10 and 100, respectively. By contrast, vB_AroA_KFSA9 achieved comparable reductions at MOIs as low as 1. Furthermore, immersion of *Salmonella*-contaminated lettuce in SalmoFresh™ at an MOI of 1000 resulted in a 2.43 log CFU/g reduction (Zhang et al., 2019). ShigaShield*™* reduced the concentration of *Shigella* on lettuce by 1.3 log and 0.6 log CFU/g when applied at MOIs of 10,000 and 1,000, respectively (Soffer et al., 2017). Likewise, commercial phage products, including SalmoFresh™, Listex™ P100, ShigaShield™, and ListShield™, typically require MOIs of 100–1000 to achieve significant bacterial reductions (Lewis and Hill, 2020). Thus, the ability of vB_AroA_KFSA9 to achieve similar reductions at MOIs as low as 1 may offer advantages from a production aspect, indicating that vB_AroA_KFSA9 is a promising candidate as a biocontrol agent for future commercial application.

Taken together, our findings demonstrate that vB_AroA_KFSA9 is a novel lytic phage with promising potential as a commercial candidate to control *A. hydrophila* on fresh produce. However, several limitations should be addressed prior to practical and commercial application. First, bacterial regrowth observed *in vitro* indicates that vB_AroA_KFSA9 may need to be used in combination with additional phages and/or hurdle approaches to achieve longer control of *A. hydrophila*. Second, long-term storage stability, large-scale production, and formulation have not yet been assessed and will be required for commercialization. Third, the efficacy of vB_AroA_KFSA9 on fresh produce was tested only under laboratory conditions. For the practical application, its performance should be validated under conditions that simulate the fresh produce chain. Thus, future studies should focus on evaluating the efficacy of vB_AroA_KFSA9 with phage cocktails and other hurdle strategies to mitigate bacterial regrowth, optimizing large-scale production and formulation, and validating its efficacy under fresh produce chain conditions to support its practical use and commercialization.

## 5 Conclusion

*A*. *hydrophila* is an emerging foodborne pathogen frequently detected in fresh produce. Despite its significance, no commercial phage products are currently available to control *Aeromonas* spp. in food. In this study, vB_AroA_KFSA9 was comprehensively characterized and demonstrated to be a novel, safe, and effective biocontrol candidate for commercial application against *A. hydrophila* in the fresh produce chain. The phage exhibited high specificity, genomic safety, novelty, and stability under various pH, temperature, and sanitizer conditions. Unlike most previous studies focusing on aquaculture applications, this study extended the applicability of vB_AroA_KFSA9 to fresh produce. Notably, significant reductions of *A. hydrophila* were achieved on cherry tomatoes and lettuce at an MOI of 1. Overall, these findings provide a strong foundation for the development of vB_AroA_KFSA9 as a safe and effective commercial biocontrol candidate to control *A. hydrophila* in the fresh produce chain. Building on this work, future studies should evaluate vB_AroA_KFSA9 within phage cocktails and hurdle strategies, and verify its performance under realistic processing and storage conditions.

## CRediT authorship contribution statement

In Young Choi: Conceptualization, Data curation, Formal analysis, Investigation, Methodology, Validation, Visualization, Writing–original draft. Su-Hyeon Kim: Data curation, Formal analysis, Investigation, Methodology, Software, Visualization, Writing–original draft. Mi-Kyung Park: Conceptualization, Project administration, Resources, Writing–review & editing, Supervision, Funding acquisition.

## Declaration of competing interest

The authors declare that they have no known competing financial interests or personal relationships that could have appeared to influence the work reported in this paper.
