# Supplementary material for: Comprehensive investigation of a novel and safe lytic phage vB_AroA_KFSA9 as a commercial candidate for biocontrol of Aeromonas hydrophila on fresh produce
Source: Curr Res Food Sci. 2025 Dec 18;12:101282. doi: 10.1016/j.crfs.2025.101282 (PMC12828811; doi:10.1016/j.crfs.2025.101282)
Supplement: Multimedia component 1 [file mmc1.pdf]

## Supplementary Materials

# Comprehensive investigation of a novel and safe lytic phage vB_AroA_KFSA9 for efficient biocontrol of *Aeromonas hydrophila* on fresh produce

**In Young Choi[1], Su-Hyeon Kim[1], and Mi-Kyung Park[1,2*]**

[1]School of Food Science and Biotechnology, and Food and Bio-industry Research Institute, Kyungpook National University, Daegu 41566, Republic of Korea

[2]Department of Infectious Disease Healthcare, Daegu 41566, Kyungpook National University, Daegu 41566, Republic of Korea

* Correspondence: parkmik@knu.ac.kr (M.-K. Park)

## Supporting Materials and Methods

### Optimization of bacterial attachment time on cherry tomato and lettuce

Cherry tomatoes and lettuce were purchased from a local grocery store. The lettuce was cut into 2 × 2 $cm^2$ pieces. The samples were soaked in a chlorine solution (200 ppm) for 10 min, rinsed three times with sterilized distilled water, and exposed to ultraviolet (UV) light for 30 min in a safety cabinet to eliminate background microorganisms (Snyder, Perry, & Yousef, 2016). Approximately 20 g of the samples were placed in separate beakers containing 200 mL of an *Aeromonas hydrophila* ATCC 7966 suspension (8 log CFU/mL) to optimize the attachment time. The fresh produce samples were incubated for 0, 15, 30, 60, 90, and 120 min in a biosafety cabinet, and then washed with PBS to detach loosely attached or unbound cells. The washed samples were transferred into sterile stomacher bags containing 180 mL of PBS and homogenized for 2 min at 120 rpm in a stomacher (Hansol Tech, Co., Seoul, Republic of Korea) for bacterial detachment and recovery. The homogenates were then plated onto *Aeromonas*-selective media containing ampicillin (HiMedia, Bombay, India) to determine the number of *A. hydrophila* attached to the surface of the samples.

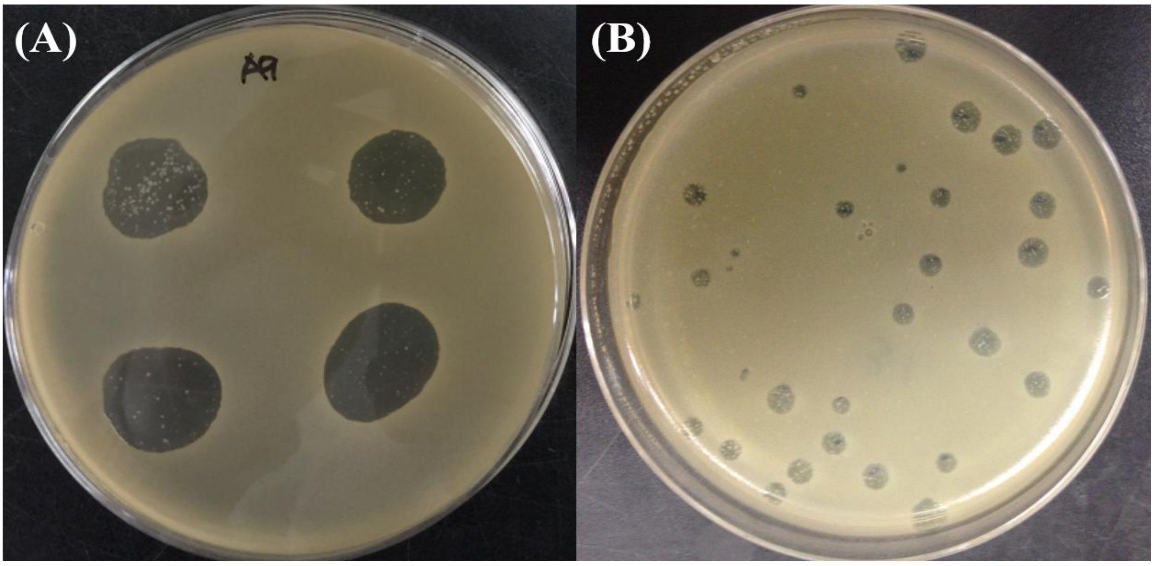


**Fig. S1.** (A) Clear zone and (B) plaques formed by vB_AroA_KFSA9.

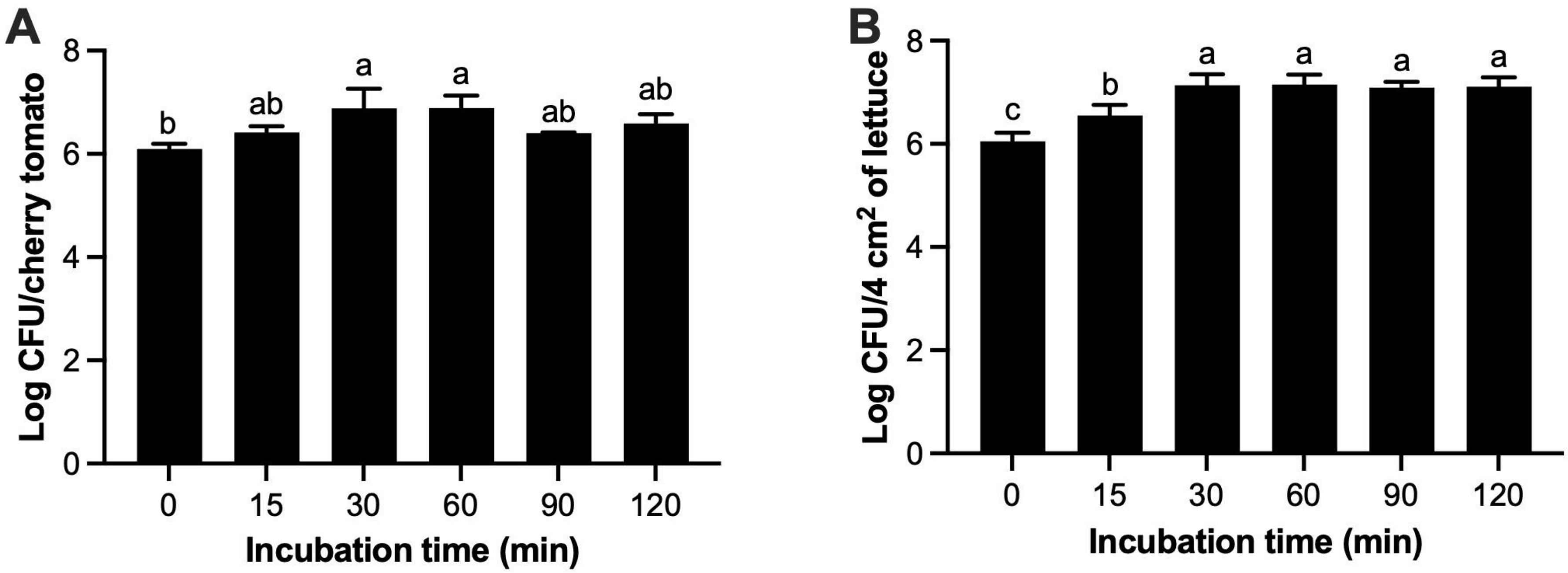


**Fig. S2.** Bacterial concentrations on (A) cherry tomato and (B) lettuce, depending on the incubation time. The letters (a–c) indicate significant differences among incubation times at $P < 0.05$ ($n = 3$, one-way ANOVA).

**Table S1.** Annotation of the vB_AroA_KFSA9 genome using the BLASTP and RAST databases.

| ORF | Functions | Category |
|---|---|---|
| 1 | Hypothetical protein | Hypothetical protein |
| 2 | Phage S-adenosyl-L-methionine hydrolase (EC 3.3.1.2) | Additional functions |
| 3 | Hypothetical protein | Hypothetical protein |
| 4 | Hypothetical protein | Hypothetical protein |
| 5 | Hypothetical protein | Hypothetical protein |
| 6 | Phage serine/threonine kinase involved in host transcription shutoff Gp0.7 | Additional functions |
| 7 | Phage DNA-directed RNA polymerase (EC 2.7.7.6) | Nucleotide metabolism and replication |
| 8 | Hypothetical protein | Hypothetical protein |
| 9 | Hypothetical protein | Hypothetical protein |
| 10 | Phage-associated ATP-dependent DNA ligase (EC 6.5.1.1) | Nucleotide metabolism and replication |
| 11 | Hypothetical protein | Hypothetical protein |
| 12 | Phage nucleotide kinase Gp1.7, phosphorylates dGMP to dGDP, and dTMP to dTDP | Nucleotide metabolism and replication |
| 13 | Hypothetical protein | Hypothetical protein |
| 14 | Host RNA polymerase inhibitor, T7-like gp2 | Nucleotide metabolism and replication |
| 15 | Phage single-stranded DNA-binding protein Gp2.5 | Nucleotide metabolism and replication |
| 16 | Phage endonuclease I (EC 3.1.21.2), four-way DNA junction-resolving | DNA packaging and phage assembly |
| 17 | Phage protein Gp2.8/Gp7.7, containing the HNH endonuclease motif | DNA packaging and phage assembly |
| 18 | Phage endolysin | Host lysis |
| 19 | Phage HNH homing endonuclease (ACLAME 27) | DNA packaging and phage assembly |
| 20 | Phage primase/helicase protein Gp4A | Nucleotide metabolism and replication |
| 21 | Hypothetical protein | Hypothetical protein |
| 22 | Phage protein Gp4.5, inhibitor of the host toxin/antitoxin system | Additional functions |
| 23 | Phage endonuclease VII | DNA packaging and phage assembly |
| 24 | Phage DNA-directed DNA polymerase (EC 2.7.7.7) | Nucleotide metabolism and replication |
| 25 | Phage DNA-directed DNA polymerase (EC 2.7.7.7) | Nucleotide metabolism and replication |
| 26 | Hypothetical protein | Hypothetical protein |
| 27 | Hypothetical protein | Hypothetical protein |
| 28 | Hypothetical protein | Hypothetical protein |
| 29 | Phage exonuclease (EC 3.1.11.3) | DNA packaging and phage assembly |
| 30 | Hypothetical protein | Hypothetical protein |
| 31 | Hypothetical protein | Hypothetical protein |
| 32 | Hypothetical protein | Hypothetical protein |

| | | |
|---|---|---|
| 33 | Phage virion assembly protein Gp7.3, ejected into infected cells | DNA packaging and phage assembly |
| 34 | Phage collar, head-to-tail connector protein Gp8 | Structure |
| 35 | Phage capsid assembly scaffolding protein Gp9 | DNA packaging and phage assembly |
| 36 | Phage major capsid protein Gp10A | Structure |
| 37 | Phage minor capsid protein Gp10A | Structure |
| 38 | Phage non-contractile tail tubular protein Gp11 | Structure |
| 39 | Phage non-contractile tail tubular protein Gp12 | Structure |
| 40 | Phage scaffold protein Gp13, required for Gp6.7 incorporation into the virion | DNA packaging and phage assembly |
| 41 | Phage DNA ejectosome component, internal virion protein Gp14 | Structure |
| 42 | Phage DNA ejectosome component, internal virion protein Gp15 | Structure |
| 43 | Phage DNA ejectosome component Gp16, internal virion protein | Structure |
| 44 | Phage noncontractile tail fiber protein Gp17 | Structure |
| 45 | Phage holin, class II Gp17.5 | Host lysis |
| 46 | Phage terminase small subunit Gp18, DNA packaging | DNA packaging and phage assembly |
| 47 | Phage Rz-like lysis protein Gp18.5, spanin protein | Host lysis |
| 48 | Phage terminase large subunit Gp19, DNA packaging | DNA packaging and phage assembly |

**Table S2.** Comparison of phages with >95.0% identity against vB_AroA_KFSA9.

| Phage | Type phage | Family | Accession | Genome length (bp) | E value | BLASTN score | Identity (%) |
|---|---|---|---|---|---|---|---|
| *Stenotrophomonas* phage IME15 | Yes | *Autotranscriptaviridae* | JX872508.1 | 38,513 | 0.0 | 331673 | 96.0 |
| *Escherichia* phage Ebrios | Yes | *Autotranscriptaviridae* | NC047942.1 | 39,752 | 0.0 | 32214 | 96.0 |
| *Aeromonas* phage PZL-Ah1 | No | *Autotranscriptaviridae* | MT681669.1 | 38,641 | 0.0 | 24258 | 96.0 |
| *Aeromonas* phage avDM11-UST | No | *Autotranscriptaviridae* | OP380607.1 | 37,561 | 0.0 | 17119 | 95.4 |

**Table S3.** Average nucleotide identity (ANI) values between vB_AroA_KFSA9 and similar phages belonging to the family *Autotranscriptaviridae*.

| Phage | Family | Genus | Accession | ANI (%) |
|---|---|---|---|---|
| *Aeromonas* phage PZL-Ah1 | *Autotranscriptaviridae* | *Teseptimavirus* | MT681669.1 | 95.1 |
| *Stenotrophomonas* phage IME15 | *Autotranscriptaviridae* | *Teseptimavirus* | JX872508.1 | 95.0 |
| *Escherichia* phage Ebrios | *Autotranscriptaviridae* | *Teseptimavirus* | NC047942.1 | 95.0 |
| *Aeromonas* phage avDM11-UST | *Autotranscriptaviridae* | *Teseptimavirus* | OP380607.1 | 93.6 |
| *Enterobacteria* phage 13a | *Autotranscriptaviridae* | *Teseptimavirus* | NC011045.1 | 76.6 |
| *Yersinia* phage YpP-Y | *Autotranscriptaviridae* | *Teseptimavirus* | NC047939.1 | 76.4 |
| *Enterobacteria* phage T7 | *Autotranscriptaviridae* | *Teseptimavirus* | NC001604.1 | 78.1 |
| *Enterobacteria* phage T3 | *Autotranscriptaviridae* | *Teetrevirus* | NC047864.1 | 74.0 |
| *Enterobacteria* phage T7M | *Autotranscriptaviridae* | *Teetrevirus* | NC047867.1 | 74.0 |
